# Visible Light Trapping against Charge Recombination in FeO_x_–TiO_2_ Photonic Crystal Photocatalysts

**DOI:** 10.3390/ma14237117

**Published:** 2021-11-23

**Authors:** Martha Pylarinou, Alexia Toumazatou, Elias Sakellis, Evangelia Xenogiannopoulou, Spiros Gardelis, Nikos Boukos, Athanasios Dimoulas, Vlassis Likodimos

**Affiliations:** 1 Section of Condensed Matter Physics, Department of Physics, University Campus, National and Kapodistrian University of Athens, 15784 Athens, Greece; pylarin@phys.uoa.gr (M.P.); alextoum@phys.uoa.gr (A.T.); sgardelis@phys.uoa.gr (S.G.); 2 Institute of Nanoscience and Nanotechnology, National Center for Scientific Research “Demokritos”, 15341 Agia Paraskevi, Greece; e.sakellis@inn.demokritos.gr (E.S.); e.xenogiannopoulou@inn.demokritos.gr (E.X.); n.boukos@inn.demokritos.gr (N.B.); a.dimoulas@inn.demokritos.gr (A.D.)

**Keywords:** photonic crystals, titanium dioxide, iron oxides, photocatalysis, slow photons

## Abstract

Tailoring metal oxide photocatalysts in the form of heterostructured photonic crystals has spurred particular interest as an advanced route to simultaneously improve harnessing of solar light and charge separation relying on the combined effect of light trapping by macroporous periodic structures and compositional materials’ modifications. In this work, surface deposition of FeO_x_ nanoclusters on TiO_2_ photonic crystals is investigated to explore the interplay of slow-photon amplification, visible light absorption, and charge separation in FeO_x_–TiO_2_ photocatalytic films. Photonic bandgap engineered TiO_2_ inverse opals deposited by the convective evaporation-induced co-assembly method were surface modified by successive chemisorption-calcination cycles using Fe(III) acetylacetonate, which allowed the controlled variation of FeO_x_ loading on the photonic films. Low amounts of FeO_x_ nanoclusters on the TiO_2_ inverse opals resulted in diameter-selective improvements of photocatalytic performance on salicylic acid degradation and photocurrent density under visible light, surpassing similarly modified P25 films. The observed enhancement was related to the combination of optimal light trapping and charge separation induced by the FeO_x_–TiO_2_ interfacial coupling. However, an increase of the FeO_x_ loading resulted in severe performance deterioration, particularly prominent under UV-Vis light, attributed to persistent surface recombination via diverse defect d-states.

## 1. Introduction

Simultaneous enhancement of light-harvesting and photogenerated charge separation has been a major requisite for the development of advanced semiconductor photocatalysts and their deployment for solar energy conversion applications using environmentally benign materials [1,2]. Augmenting light utilization has been intensely pursued by rational materials’ design [3], especially for the benchmark titania (TiO_2_) photocatalysts [4,5], ranging from electronic bandgap and defect engineering by doping [6,7], compositing with plasmonic [8,9], narrow bandgap semiconducting [10,11] as well as graphene-based [12,13] nanomaterials. A promising, though the challenging approach to intensify light-matter interactions and amplify photon capture is based on the fabrication of photocatalytic photonic crystals (PCs), i.e., periodically ordered structures, whose refractive index is spatially modulated on the scale of light’s wavelength [14,15,16,17]. PC-assisted photocatalysis has been accordingly put forward as an advanced structural modification that may selectively improve light trapping at frequencies of weak materials’ electronic absorption, by retarding light propagation at the edges of the fundamental photonic bandgap (PBG) [18] and higher frequency photonic bands [19] as well as by multiple light scattering due to the persistent disorder and imperfections in PCs [20]. Besides extending the optical path of incident photons, PC structures, the most common being inverse opals as well as periodically ordered alumina and GaN structures tailored by electrochemical anodization [21,22,23], provide a macroporous scaffold that along with the secondary mesoporosity of its inorganic skeleton lead to a network of interconnected macro-mesopores [24], which facilitate reactants adsorption and diffusion during the photocatalytic process [25]. Substantial research has been thus devoted to exploiting these advantageous characteristics in combination with materials’ compositional properties in order to develop visible light-activated (VLA) photonic photocatalysts [26], including coupling PCs with plasmonic [27,28,29,30] and graphene nanomaterials [31,32,33] as well as metal-oxide (MO) nanoclusters [34,35,36].

In particular, titania’s surface modification by nanoscale MOs has been attracting significant attention as a versatile method for solar light photocatalytic applications via MOs’ action as VLA sensitizers that also promote charge separation via interfacial charge transfer [37,38,39,40,41] and/or co-catalysts of TiO_2_ [42,43,44]. Among the different MOs, nanostructured iron oxides, especially hematite α-Fe_2_O_3_, have been drawing continuous interest for VLA photocatalysis due to their ample earth abundance, narrow bandgap, nontoxicity, and low cost [45]. Despite limitations due to the inherently poor electrical conductivity, short diffusion lengths, and fast recombination that impair the photocatalytic activity of iron oxides (FeO_x_) [46], compositing with titania has shown promising results for organic pollutant decomposition and water splitting under favorable FeO_x_–TiO_2_ interfacial coupling that enables efficient charge separation [47,48,49,50,51,52], especially under visible light [53,54]. Composite Fe_2_O_3_/TiO_2_ semiconductors under UV and/or visible light, may cause interparticle charge transfer from TiO_2_ to Fe_2_O_3_ nanoparticles since both the valence and conduction bands of ferric oxides to lie within titania’s bandgap [55], frequently leading to performance deterioration for the coupled system because of the weaker photocatalytic activity of Fe_2_O_3_. Controlled grafting of nanoscale Fe_2_O_3_ clusters on titania’s surface has been performed by Tada et al. [56] employing iron acetylacetonates’ chemisorption on TiO_2_ followed by post-calcination [57], featuring the chemisorption-calcination-cycle (CCC) technique that was successfully applied for the deposition of various MOs on TiO_2_ [37]. Thorough investigations of Fe_2_O_3_−surface-modified TiO_2_ have shown that high photocatalytic activity could be attained via Fe–O–Ti interfacial bonds inducing d-surface states, which raise the valence band of titania circumventing the unfavorable thermodynamics for the Fe_2_O_3_−TiO_2_ system. Moreover, deposition of isolated Fe(III)-oxo clusters by atomic layer deposition (ALD) [53] and recently by wet impregnation and post-thermal decomposition of Fe acetylacetonate precursors onto TiO_2_ [54], resulted in enhanced VLA reactivity for organic dye degradation and the oxygen evolution reaction during water splitting, respectively. Decoration of high-quality ALD-fabricated TiO_2_ inverse opals by Fe_2_O_3_ nanoparticles using a post-hydrothermal treatment resulted in substantial improvement of the photocurrent density under UV-vis illumination related to the enhanced visible light absorption and reduced electron-hole recombination in Fe_2_O_3_−TiO_2_ [58]. Very recently, surface modification of TiO_2_ PC films by CoO_x_ nanoclusters using the CCC method led to a marked performance amplification for organics photocatalytic decomposition at low CoO_x_ loadings associated with the beneficial combination of slow-photon-assisted visible light trapping with the increased charge separation in CoO_x_−TiO_2_ inverse opal films [59].

In this work, surface deposition of nanoscale FeO_x_ on TiO_2_ PC films is systematically investigated in order to exploit synergistic effects of PC-assisted visible light trapping and improved charge separation in FeO_x_–TiO_2_ photocatalytic films. TiO_2_ inverse opal films were deposited by the co-assembly of variable diameter colloidal sphere templates and a hydrolyzed TiO_2_ precursor and then surface modified by successive CC cycles using Fe(III) acetylacetonate, which allowed the controlled variation of FeO_x_ loading on PBG engineered TiO_2_ PCs. Deposition of low amounts of FeO_x_ nanoclusters on TiO_2_ inverse opals resulted in the selective improvement of photocatalytic degradation of salicylic acid and photocurrent density under visible light, surpassing similarly modified P25 films and evading the loss of holes oxidizing ability that is usually observed in VLA TiO_2_ photocatalysts. The observed enhancement was related to the synergy of light trapping and surface states induced by the interfacial coupling between TiO_2_ and FeO_x_. However, increasing iron oxide loading competed with the photonic enhancement, especially under UV-Vis illumination due to the persistent surface recombination by defect d-states.

## 2. Materials and Methods

### 2.1. Materials Fabrication

**Scheme 1:**
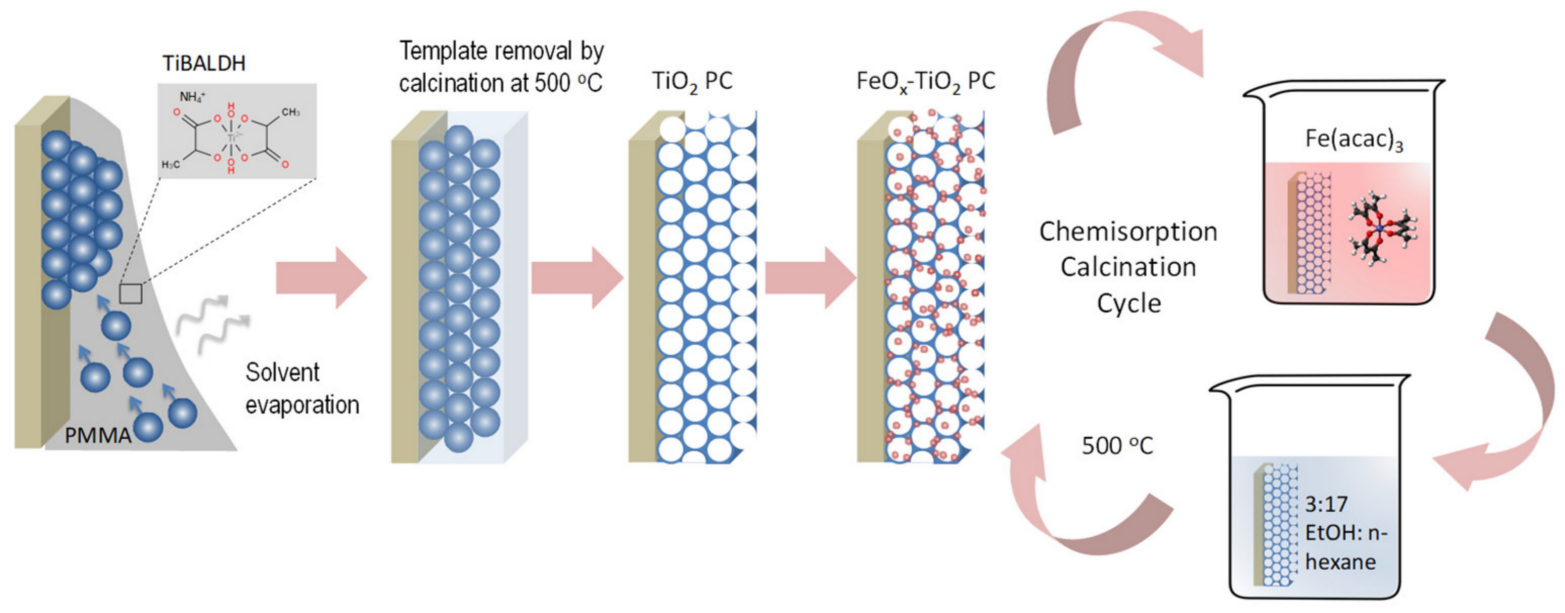
Deposition process for co-assembled FeO_x_–TiO_2_ inverse opals.

TiO_2_ inverse opals of variable PBGs were fabricated by the convective evaporation-induced co-assembly of polymer microspheres with titanium(IV) bis(ammonium lactato)dihydroxide (TiBALDH) sol-gel precursor (Scheme 1) [60,61].

Specifically, colloidal dispersions of monodisperse polymer spheres of different diameters, namely 220 nm polystyrene (PS) spheres (Polysciences Inc., 8% CV, SD = 0.02 μm, 2.7 % solids *w*/*v*) and poly (methyl methacrylate)-PMMA spheres with diameters of 260 nm (Microparticles GmbH, 2.4% CV, SD = 0.006 μm, 5.0 % solids *w*/*v*) and 330 nm (Polysciences Inc., <5% CV, SD = 0.019 μm, 2.5% solids *w*/*v*), were used to tune the photonic films PBG across the FeO_x_–TiO_2_ electronic absorption edge. Glass slides cleaned in Hellmanex (Hellmanex III, Helma Analytics, Müllheim, Germany), followed by ultrasound sonication in acetone and ethanol (EtOH), were nearly vertically suspended in a beaker containing 10 mL of 0.1 wt.% dilute sphere suspension and 0.056 mL of fresh titania precursor prepared by mixing 0.25 mL of 50 wt.% aqueous TIBALDH solution (388165, Sigma-Aldrich, St. Louis, MO, USA), 0.5 mL of 0.1 M HCl, and 1 mL EtOH. The beakers were kept at 55 °C until the solvent fully evaporated, leading to self-assembled films containing the titania gel within the opal interstices. The films were then calcined at 500 °C for 2 h in the air (1 °C/min), to remove the colloidal template and crystallize titania inverse opals. The pristine TiO_2_ PC films were labeled as PC220, PC260, and PC330 after the templating sphere diameter.

Surface modification of the titania inverse opal by iron oxides was implemented using as precursor salt the iron(III) acetylacetonate complex, Fe(acac)_3_ (12534, Alfa Aesar Thermo Fisher Scientific, Heysham, Lancashire, UK) for the CCC technique (Scheme 1). The PC films were immersed for 24 h at room temperature in a volatile 6.5 × 10^−4^ M Fe(acac)_3_ solution prepared from 23 mg Fe(acac)_3_ diluted in 100 mL of solvent consisting of 15 mL EtOH and 85 mL normal hexane (ethanol: n-hexane = 3:17 *v*/*v*) [56]. Copious rinsing of the impregnated films with the solvent was used to remove physisorbed Fe(acac)_3_ molecules, followed by calcination in the air (1 °C/min) at 500 °C for 1 h to remove organic species and allow the formation of stable FeO_x_–TiO_2_ composites via Fe-O-Ti interfacial bonds [57]. The CC treatment was repeated up to three times for the best performing PC260 inverse opals. The corresponding films were labeled as FeO_x_-PCXXX-nth, where XXX = 220, 260, and 330 is the template sphere diameter and n is the CC cycle index.

Mesoporous titania films were spin-coated on cleaned glass substrates using a paste of titania nanopowder (Aeroxide^®^ P25, Evonik, Essen, Germany) [59] as a benchmark reference to validate the photonic films’ activity. After post-annealing at 450 °C in the air to remove organic species, the P25 films were surface modified using Fe(acac)_3_ under identical conditions, and the corresponding films were labeled as FeO_x_-P25-nth according to the CCC cycle index.

### 2.2. Characterization

The materials’ morphological and structural properties were investigated by an FEI scanning electron microscope (SEM) (Quanta Inspect, FEI, Eindhoven, The Netherlands) equipped with an energy-dispersive X-ray analyzer (EDX) (EDXDX4, Mahwah, NJ, USA). The structure and phase composition of the PC films were studied by a transmission electron microscope (TEM, CM20, FEI, Eindhoven, The Netherlands) coupled with energy-filtered TEM (GIF 200, Gatan, Pleasanton, CA, USA) and an FEI Talos F200i field-emission scanning transmission electron microscope (Thermo Fisher Scientific Inc., Waltham, MA, USA) operating at 200 kV, equipped with a windowless energy-dispersive spectroscopy microanalyzer (6T/100 Bruker, Hamburg, Germany). The films’ structural properties were also investigated by micro-Raman spectroscopy (inVia Reflex, Renishaw, London, UK) using the 514 nm line of an Ar^+^ ion laser. The laser beam was focused onto the films by a ×100 objective (NA = 0.90), while the laser power was controlled at low-density levels (<0.1 mW/μm^2^) to evade local heating. X-ray photoelectron spectroscopy (XPS) was performed on a PHOIBOS 100 (SPECS, Berlin, Germany) hemispherical analyzer using Mg-Kα (1253.6 eV) X-ray source (SPECS XR50). The spectrometer was calibrated on clean Ag, Cu, and Au samples for which the Ag 3d5/2, Cu 2p3/2, and Au 4f7/2 peak positions were determined at 368.3, 932.7, and 84 eV, respectively. The XPS spectra were collected at a 52° take-off angle using a pass energy of 7 eV. The adventitious C 1s set to 284.8 eV was used for charge referencing. Fitting was performed using XPS Peak Fit and Sirley background subtraction. Photoluminescence (PL) measurements were carried out using the focused beam of a 275 nm light-emitting diode as excitation, while the PL signal was collected through a long-pass 320 nm filter by a fiber optic spectrometer (LR1, ASEQ Instruments, Vancouver, Canada). The optical properties were investigated by diffuse and specular reflectance measurements using a fiber-optic diffuse and a 15° specular reflectance accessory on a Cary60 UV-Vis spectrometer, respectively. A Halon reference and a UV-enhanced Al mirror were used for background determination. Attenuated total reflection Fourier-transform infrared (ATR-FTIR) spectra were recorded on a JASCO 470Plus (JASCO Corp., Hachioji, Tokyo, Japan) FT-IR spectrometer equipped with a PIKE MIRacle ATR accessory using a single reflection diamond/ZnSe crystal plate.

Photoelectrochemical measurements were performed in a three-electrode configuration using a CS350 potentiostat/galvanostat (Wuhan Corrtest Instruments Corp., Ltd., Wuhan, China), Ag/AgCl as a reference, and a Pt foil as counter electrodes. The working photoelectrode was prepared by depositing the PC and P25 films on cleaned fluorine-doped tin oxide (FTO) conductive glass substrates (7 ohms/sq, Sigma-Aldrich, St. Louis, MO, USA) followed by FeO_x_ surface modification using the CCC method. The electrolyte was 0.5 M NaOH, while UV–Vis and illumination were provided by a 300 W Xe lamp (120 mW/cm^2^) combined with a long pass 400 nm cutoff filter for visible light selection. Electrochemical impedance spectroscopy (EIS) measurements were performed at an open-circuit voltage (V_OC_), in the 10^4^–10^−1^ Hz frequency range with 10 mV ac amplitude.

### 2.3. Photocatalytic Performance

Photocatalytic activity evaluation for the PC and P25 films was carried out on the aqueous phase degradation of salicylic acid (SA) under UV–Vis and Vis illumination. Films of 2 cm^2^ area were initially stirred in vials containing 3 mL of 30 μΜ SA aqueous solution for 30 min under dark in order to reach adsorption-desorption equilibrium. The solution pH was fixed at 3 by dilute HCL in order to enhance SA adsorption on the TiO_2_ films [33]. A 150 W Xe lamp was used as a UV–Vis light source along with a heat-reflective mirror (20CLVS-3, Newport, Irvine, CA, USA), while visible light was selected by a 400 nm cutoff filter. The horizontal light beam was directed on the films using a UV-enhanced Al mirror at 100 mW/cm^2^ power density. Aliquots of 0.5 mL were periodically taken out from the SA solution and analyzed in the spectrophotometer using a 10 mm path length quartz microcell. Additional photocatalytic tests were performed in the presence of 5 μM isopropanol (IPA) and formic acid as hydroxyl radical and hole scavengers, respectively. The photocatalytic tests were repeated three times and standard errors were derived for the mean kinetic constants.

## 3. Results and Discussion

### 3.1. Structural and Optical Properties

**Figure 1:**
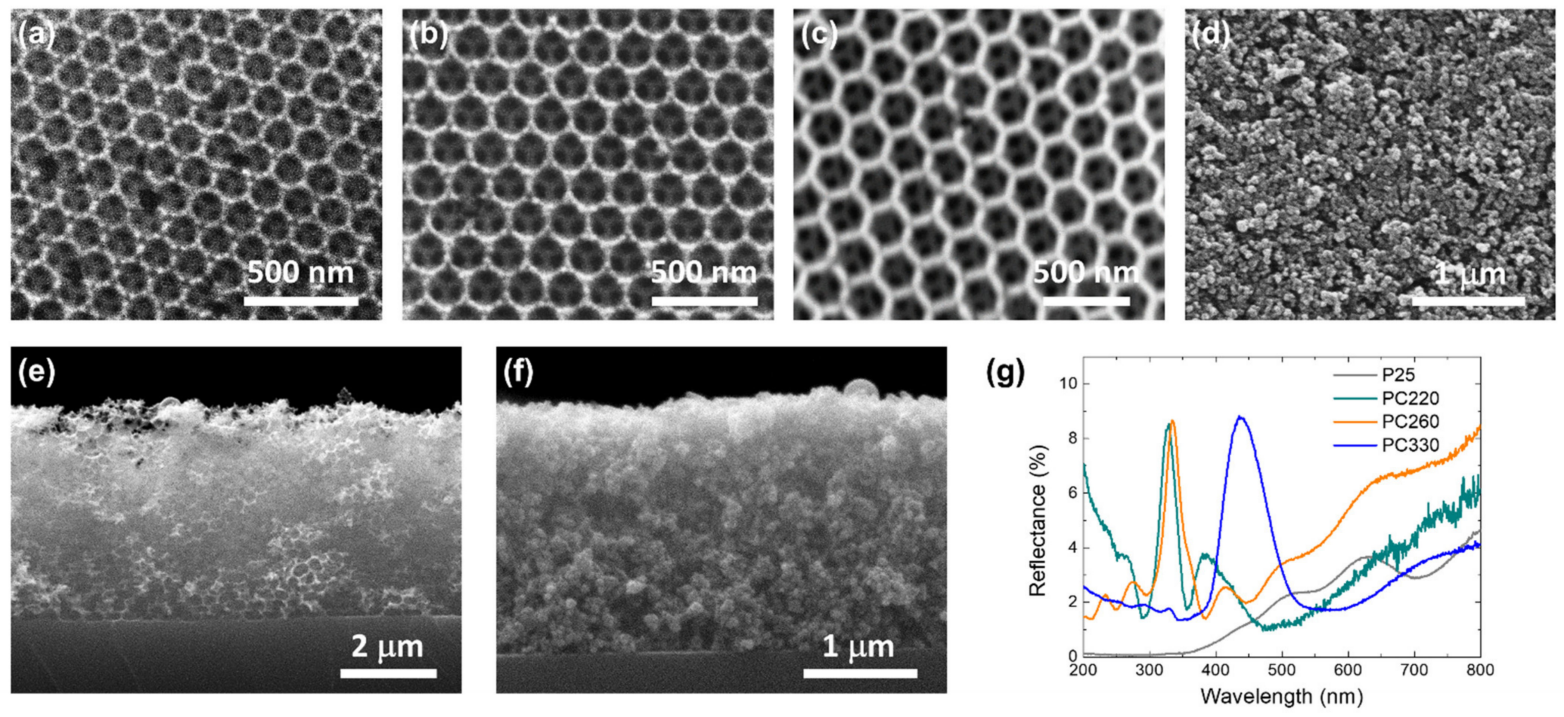
Top-view SEM images of (**a**) PC220, (**b**) PC260, (**c**) PC330 TiO_2_ inverse opals in comparison with the (**d**) mesoporous P25 films. Cross-sections of (**e**) PC260 and (**f**) P25 films. (**g**) Specular reflectance (R%) of the PC and P25 films at 15° incidence angle.

Co-assembly of the TIBALDH precursor with the sacrificial colloidal polymer spheres resulted in the formation of well-ordered inverse opal structures consisting of the (111) planes of an fcc crystal lattice of void spheres, as shown by the SEM images of Figure 1a–c, distinctly different from the rough, aggregated morphology of mesoporous P25 films (Figure 1d). The hexagonally ordered macropores of the inverse opals structure were interconnected through smaller ones (dark areas within the large spherical voids) that form after calcination at the contact points of neighboring polymer spheres facilitating mass transfer in the pore network. Their mean diameter (*D*) determined by SEM, increased proportionally to the colloidal sphere size (Table 1), while the obtained values were about 60% smaller than those of the templating spheres because of the persistent volume contraction of crystalline metal oxide inverse opals [33,60]. Cross-section SEM images revealed that the thickness of the inverse opal films was approximately 4 μm (Figure 1e), while that for the P25 film was approximately 2.2 μm (Figure 1f). The P25 films will accordingly have considerably higher, by ca. 50%, titania mass loading compared to the photonic ones, assuming identical wall mesoporosity and an ideal solid filling factor of 0.26 for the inverse opals. The presence of PBG was identified by specular reflectance (R%) measurements for all the photonic films at 15° incidence (Figure 1g). A clear R% peak due to Bragg reflection was invariably observed for all PCs at wavelengths increasing with the inverse opal diameter (Table 1), characteristic for the incomplete PBG (stopband) formation along the [111] direction in titania inverse opal structures [26].

The stopband variation for the different inverse opals contrasts the Fabry-Pérot interference fringes most clearly observed for the mesoporous P25 films at wavelengths exceeding titania’s electronic absorption edge at λ > 400 nm. It should be noted that the amplitude of the Bragg peak was relatively low (<10%) for all co-assembled photonic films reflecting the presence of submillimeter-sized PC domains interrupted by cracks within the coarse spot of the measuring beam (1–2 mm^2^) used in the R% measurements [32], which lead to enhanced diffuse scattering especially toward the low-energy PBG edge (vide infra) and possibly the increase of R% below 260 nm, most prominent for PC220. Applying modified Bragg’s law for first-order diffraction from the (111) fcc planes (Supplementary material S1) yielded the effective refractive index $n_{eff}$ and the corresponding solid filling fraction 1−f (f = 0.74 for the fcc lattice) in the air (Table 1) based on the experimental λ_exp_ (15°) values and the inverse opal diameters *D*. The obtained values for 1−f were appreciably smaller than the theoretical one of 0.26 for complete filling of the inverse fcc lattice, corroborating the formation of mesoporous nanocrystalline walls in the co-assembled TiO_2_ inverse opal [62]. Furthermore, using the derived filling fractions and the water refractive index ($n_{H_{2}O}=1.33$), the PBG positions were estimated for the inverse opals in water (Table 1), where the aqueous phase photocatalytic reaction is carried out. 

Surface modification of the TiO_2_ films by successive CC cycles of Fe(acac)_3_ had no observable effect either on the PCs’ periodicity and macropore diameter or the P25 film morphology, as shown in Figure S1. The deposition of Fe species was verified by EDX elemental analysis using the Ti K and Fe K peaks for the CCC surface-modified TiO_2_ films. Quantification indicated that the Fe loading amount increased continuously with the number of cycles, as shown in Table S1. Comparison between the different photonic films showed that the PC220 films presenting the smallest macropore diameter exhibited slightly higher Fe (at%) uptake. This complies with the higher surface area and mesopore volume reported for co-assembled TiO_2_ PC films with small macropores increasing the available interfaces between skeletal walls [59]. 

**Figure 2:**
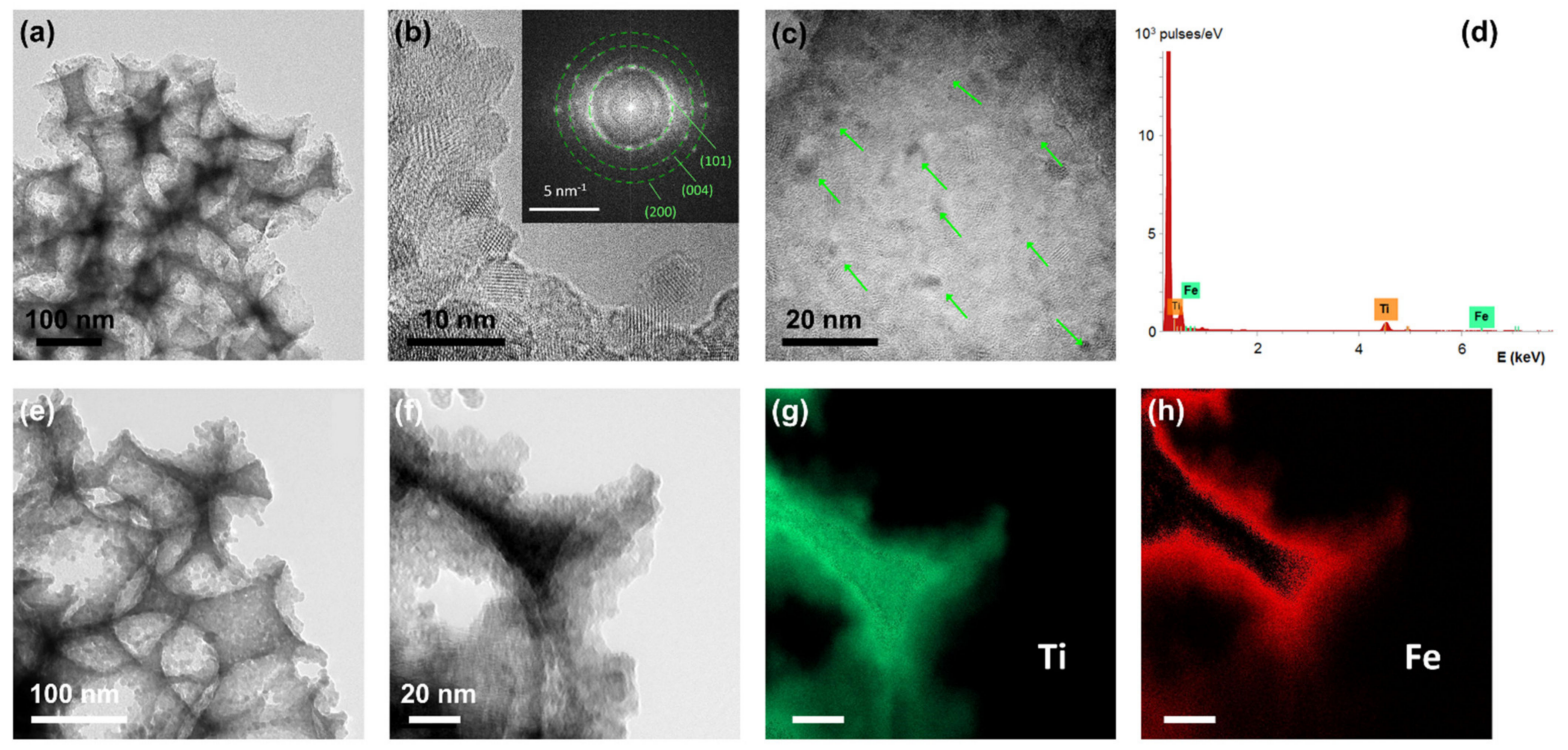
(**a**–**c**) TEM images for FeO_x_-PC260-1st at different magnifications. The inset in (**b**) shows the corresponding FFT pattern (**d**) Local EDX spectrum corresponding to the image (**b**). (**e**,**f**) TEM images for FeO_x_-PC260-3rd at different magnifications and the (**g**) Ti and (**h**) Fe EFTEM elemental maps corresponding to (**f**).

The phase composition of the surface-modified inverse opals was studied by TEM, as shown in Figure 2. TEM images for FeO_x_-PC260-1st (Figure 2a–c) showed the presence of mesoporous walls consisting of aggregated nanoparticles (<10 nm) in the anatase TiO_2_ phase. The latter was identified by the most intense diffraction spots from the (101), (004), (200) planes with corresponding d-spacings of 0.35, 0.24, and 0.19 nm, as shown by the fast Fourier transform (FFT) patterns in the inset of Figure 2b. Moreover, dark inclusions of 1–3 nm could be discerned among the anatase nanoparticles, with no distinct FFT pattern that could be related to iron oxides. These nanoscale deposits, whose crystallinity could not be resolved, point to the presence of ultrafine FeO_x_ species, complying with a recent thorough study on the immobilization of MO co-catalysts, including FeO_x_, on TiO_2_ using the Fe(acac)_3_ precursor [44]. Moreover, local EDX spectral analysis in the TEM image of FeO_x_-PC260-1st (Figure 2c) verified the presence of Fe species, as shown in Figure 2d. Energy filtered TEM (EFTEM) analysis was additionally carried out in order to trace the location and distribution of FeO_x_ species on the nanocrystalline anatase walls of the FeO_x_-PC260-3rd films with the highest Fe loading amount (Figure 2e–h).

Ti and Fe elemental maps obtained for the area depicted in the TEM image of Figure 2f showed the decoration of the TiO_2_ inverse opal skeleton with Fe species, indicating that both dispersed FeO_x_ nanoclusters, as well as their aggregates, are deposited in FeO_x_-PC-3rd films.

**Figure 3:**
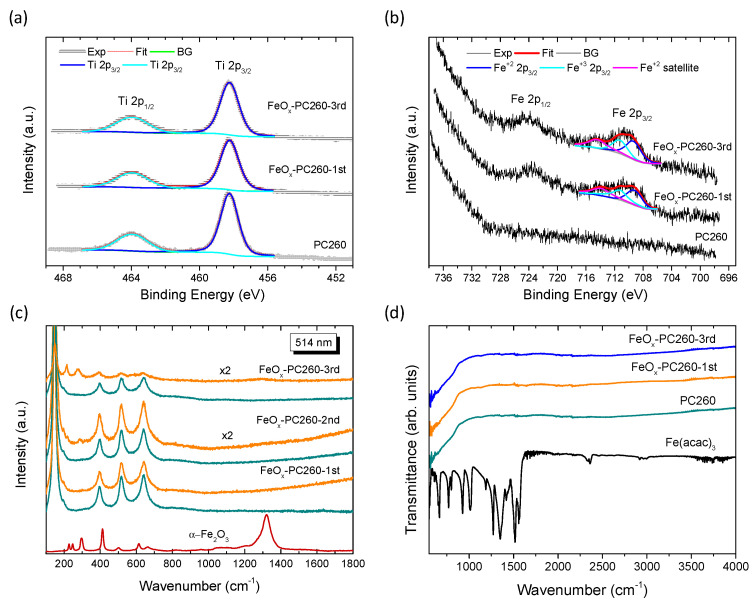
(**a**) Ti 2p and (**b**) Fe 2p core level for the pristine and surface modified FeO_x_–PC260 films after the 1st and 3rd CC cycle. Solid lines correspond to the total fit and the individual peaks of the Ti 2p and Fe 2p_3/2_ spectral deconvolution. (**c**) Raman spectra of the FeO_x_–PC260 films for the different CC cycles before (green) and after (orange) laser annealing in comparison with a reference hematite spectrum at 514 nm. (**d**) ATR-FTIR spectra of the neat Fe(acac)_3_ precursor, PC260, and FeO_x_–PC260 after the 1st and 3rd CC cycles.

The surface composition of the FeO_x_–PC films was further investigated by XPS. Figure 3a shows the characteristic Ti 2p spin-orbit doublets for the pristine and FeO_x_-PC260 inverse opals after the 1st and 3rd CC cycle. Close fitting of the experimental peaks could be obtained for both pristine and FeO_x_–modified PC260 films using two distinct components at 458.23 eV with full-width at half-maximum (FWHM) of ~1.3 eV and 463.93 eV with FWHM~2.1 eV, respectively. The Ti 2p_1/2_–Ti 2p_3/2_ splitting was 5.7 eV, indicating the presence of Ti^4+^ ions and stoichiometric TiO_2_ [63] with no observable contribution of lower Ti oxidation after surface modification with Fe(acac)_3_. Moreover, the Fe 2p_3/2_ and 2p_1/2_ core level peaks in Figure 3b verified the formation of iron species in the FeO_x_—modified PC260 films. The Fe 2p_3/2_ peak curve fitting showed contributions from both Fe^+2^ and Fe^+3^ species at 709.3 eV and 711.2 eV respectively. The Fe^+2^ satellite peak was also identified at 714.4 eV, about 5.1 eV above the Fe^+2^ main peak as expected for ferrous compounds [64]. The presence of both Fe^2+^ and Fe^3+^ oxidation states is accordingly inferred for the FeO_x_–modified PC films, in agreement with previous reports [44,56], with a significant contribution from Fe^2+^ oxides such as FeO and Fe(OH)_2_, which are known to be prone to non-stoichiometry and oxidation, respectively [65].

The structural and compositional properties of the photonic and P25 films before and after FeO_x_ modification were further studied by Raman spectroscopy at 514 nm excitation. The Raman spectra for the bare PC films presented the characteristic vibrational modes of nanocrystalline anatase TiO_2_ at approximately 147 (E_g_), 198 (E_g_), 399 (B_1g_), 518 (A_1g_ + B_1g_), and 641 cm^−1^ (E_g_) [66], as shown in Figure S2. No Raman bands due to polymer and carbonaceous species or other TiO_2_ phases could be detected, indicating the growth of single-phase anatase TiO_2_ PC films after thermal treatment at 500 °C. Substantial blue-shifts and spectral broadening of the Raman bands though, were detected, especially for the most intense low-frequency mode, compared to the P25 films (inset of Figure S2).

This behavior can be associated with the breakdown of the *q* = 0 selection rule and the presence of sub-10 nm anatase nanocrystallites in the TiO_2_ photonic films [66], in perfect agreement with the high resolution-TEM analysis and recent Raman results corroborating the partial inhibition of anatase growth in co-assembled TiO_2_ inverse opals using the TiBALDH precursor [32,59].

No additional Raman bands due to iron oxides could be traced after CCC modification by Fe(acac)_3_ on either the PC (Figure 3c) or P25 (Figure S3) films, even though excitation at 514 nm (2.41 eV) approaches closely the absorption edge of most iron oxides [65] and thus it is expected to be highly sensitive to the films’ surface composition by means of resonance Raman scattering [47]. To explore the presence of FeO_x_ on the CCC modified films, local annealing experiments were performed by focusing the 514 nm laser beam on the FeO_x_–PC260 (Figure 3c) and FeO_x_–P25 (Figure S3) films by the 100× objective at full laser power (2.5 mW) for 60 s in order to investigate the local oxidation of FeO_x_ nanoclusters to stable iron oxide phases. Normal Raman spectra were then recorded on the same spot using low laser power density (0.05 mW/μm^2^) to trace any local phase transformation. A series of new Raman peaks indeed emerged at approximately 215, 277, and 1310 cm^−1^ for the photonic FeO_x_−PC260-2nd and FeO_x_–PC260-2nd films (Figure 3c) and from the first CC cycle for FeO_x_−P25 (Figure S3). These Raman bands are close to the characteristic Raman modes of hematite *a*-Fe_2_O_3_ [67], as shown in the reference spectra of Figure S4a [68]. They could be identified during laser heating experiments on maghemite (γ-Fe_2_O_3_) nanoparticles, as shown in Figure S4b, while the same laser-induced transformation to hematite has been previously reported for the less crystalline ferrihydrite and wüstite phases [67,69]. These results suggest that poorly crystallized or even amorphous FeO_x_ nanoclusters are deposited on the anatase walls of the surface-modified PC films, consistent with the TEM-EDX and XPS results. This was further corroborated by comparative ATR-FTIR spectra on FeO_x_–PC260 films with respect to the individual constituents, as shown in Figure 3d. No observable IR mode from the different iron oxides and oxyhydroxides [65] could be traced in the studied frequency range (550–4000 cm^−1^) after thermal treatment at 500 °C. It should be noted that Fe(II) oxides i.e., FeO, Fe(OH)_2,_ and the mixed-valence Fe_3_O_4_ are not expected to produce strong IR bands in this spectral range [65], especially when these oxides are poorly crystallized. These results comply with the recent report on the amorphous state of MO_x_ species on surface-modified TiO_2_ by wet impregnation-post calcination of metal acetylacetonate precursors, regardless of the calcination protocol [44].

**Figure 4:**
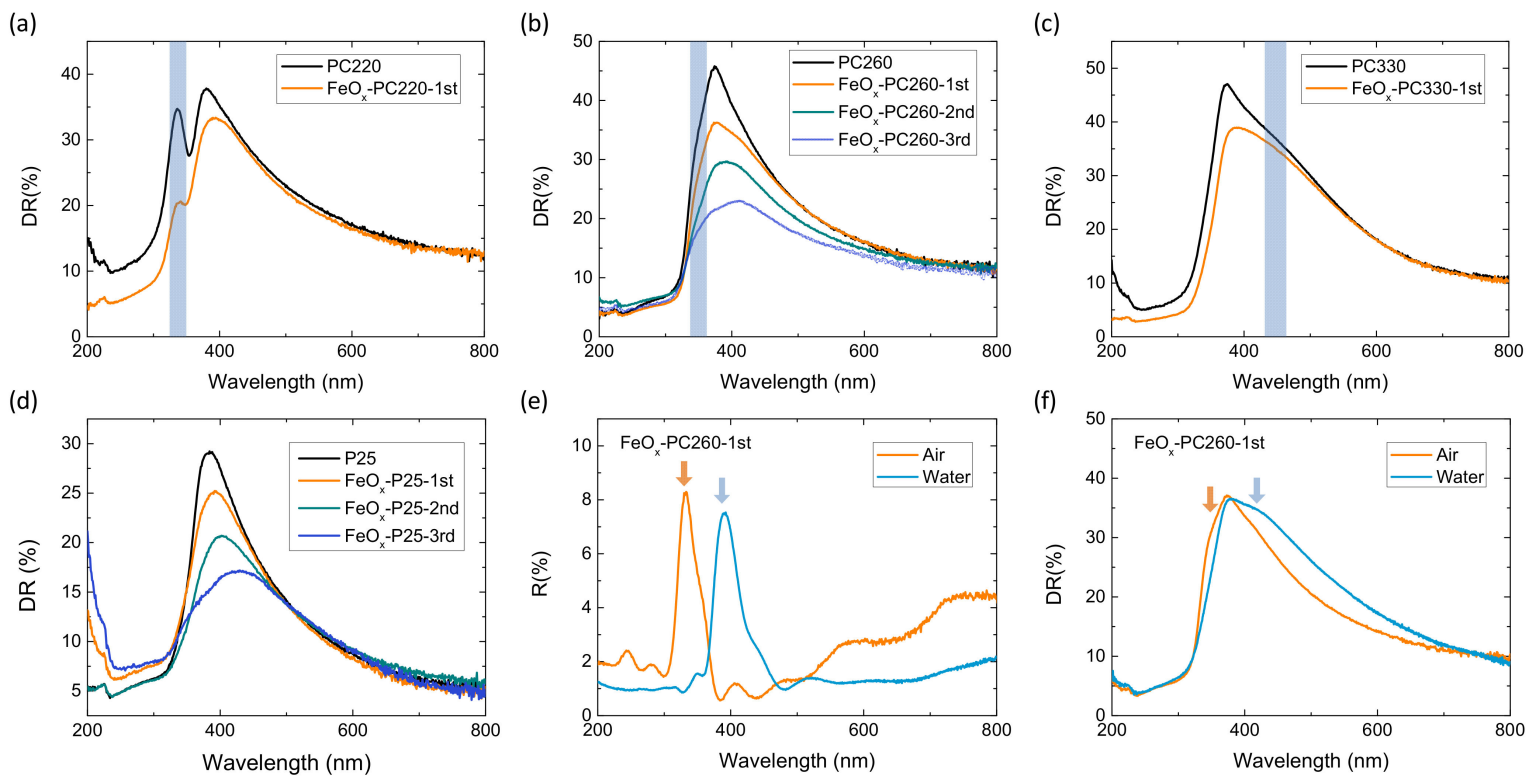
Diffuse reflectance spectra for the (**a**) PC220, (**b**) PC260, (**c**) PC330, and (**d**) P25 films before and after FeO_x_ CCC modification. Shaded bands denote the positions of the DR% Bragg reflectance for the PC films. Comparative (**e**) diffuse and (**f**) specular reflectance spectra for FeO_x_–PC260-1st films in air and water.

Figure 4 compares the diffuse reflectance spectra of the FeO_x_—modified inverse opal and mesoporous titania films. An intense DR% band was clearly observed at 336 nm for PC220, while broader ones were resolved at about 350 and 455 nm for PC260 and PC330, respectively. These bands followed closely the corresponding Bragg R% peaks of the PC films (Figure 1g), while they were distinctively different from the absorption edge of anatase at 375 nm and the corresponding DR% spectrum of P25 films (Figure 4d), whose absorption edge was red-shifted by about 20 nm with respect to the PC films due to small amounts of rutile nanoparticles that possess a lower (~3.0 eV) bandgap. Moreover, the DR% bands of the PC films were systematically red-shifted by 6–10 nm with respect to the corresponding *λ*(0°) values (Table 1). This behavior can be related to the enhanced diffuse scattering at the low-energy (red) edge of the PC stop band, where slow photons localize mainly in the high refractive index anatase inverse opal frame [32]. Surface modification by FeO_x_ nanoclusters resulted in the decrease of DR% intensity below 500–600 nm, most clearly detected for the FeO_x_–PC260 and FeO_x_–P25 films that were subjected up to three CC cycles. The corresponding FeO_x_–P25 absorbance spectra derived from the Kubelka-Munk transform with no interference from Bragg reflection (Figure S5), showed the progressive extension of electronic absorption for FeO_x_–TiO_2_, reaching 500 nm after the 3rd CC cycle, due to the contribution of iron oxides. This effect was likewise observed for the CCC-modified PC films together with the continuous drop of stopband reflection arising from the increased FeO_x_ visible light absorption.

### 3.2. Photocatalytic Activity

**Figure 5:**
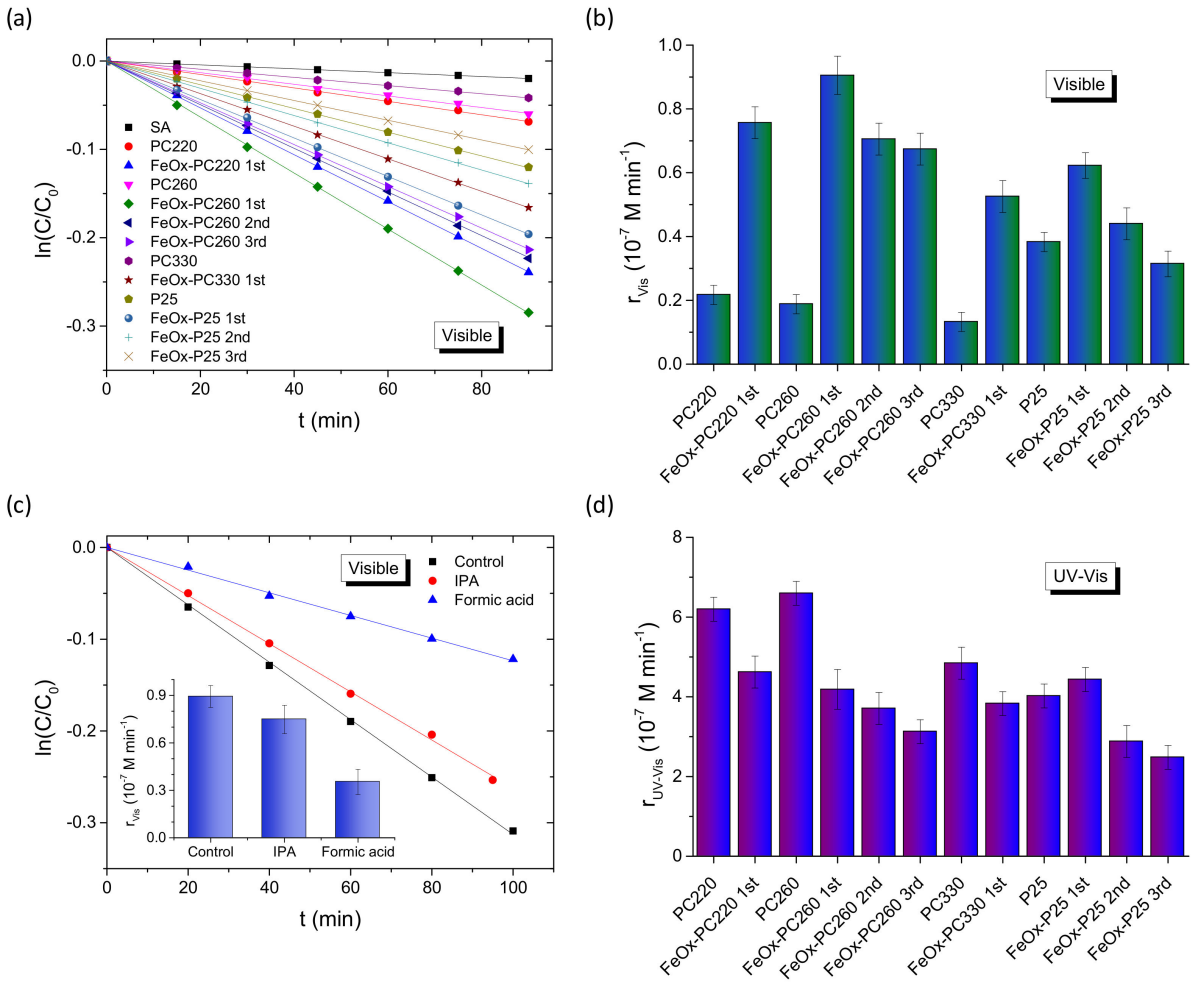
(**a**) SA photodegradation kinetics for the FeO_x_–modified PC and P25 films under visible light and (**b**) the corresponding reaction rates *r*_Vis_. (**c**) SA degradation kinetics for FeO_x_–PC260-1st in the presence of IPA and formic acid as hydroxyl radical and hole scavengers, respectively. (**d**) The reaction rates *r*_UV-Vis_ for the FeO_x_−modified PC and P25 films under UV-Vis light.

The photocatalytic performance evaluation for the FeO_x_—modified PC and P25 films was carried out on the degradation of SA, a colorless water pollutant, which, unlike dyes, absorbs at λ < 330 nm (Figure S6a), well below the PCs’ stop bands in water, and thus precludes any photonic enhancement by means of slow photon spectral overlap with the SA absorbance [32]. In addition, the photocatalytic tests were performed at acidic pH = 3, which favors chemisorption of SA molecules on titania and their preferential oxidation by valence band holes [33,70], providing a first indication for the oxidation power of visible light-generated holes in the FeO_x_–TiO_2_ films. Control experiments in the absence of either PC or P25 films, indicated weak SA degradation under both visible and UV–Vis light, while the continuous decrease of the SA concentration (*C*) was observed with time in the presence of the photocatalytic films, as shown in Figure S6b,c. For all samples, the ln(*C*/*C*_0_) vs. *t* plots, with *C*_0_ being the initial SA concentration after dark adsorption, varied linearly with time under both visible (Figure 5a) and UV-Vis light (Figure S6d), indicating that SA photodegradation followed pseudo-first-order kinetics. The apparent kinetic constants *k*_Vis_ and *k*_UV-Vis_ were accordingly determined from the slopes of the linear ln(*C*/*C*_0_) vs. *t* plots and the corresponding reaction rates were then calculated as *r* = *kC*_0_ for low (<mM) pollutant concentrations [32] in order to determine the films’ activity independently of *C*_0_ variations due to SA adsorption. The PC220 and PC260 films showed rather weak SA photodegradation under visible light that decreased further for PC330. This small VLA photocatalytic activity can be related to either unintentional doping effects in the pristine PCs and/or weak UVA radiation leakage from the filtered Xe lamp source in combination with slow light trapping effects at the anatase absorption edge, which have been amply identified for TiO_2_ PCs under UV-Vis light [18,26].

Specifically, TiO_2_ inverse opals with low-energy stopband edges approaching titania’s electronic absorption edge present marked improvements of the photocatalytic rates due to red-edge slow-photons, while detrimental Bragg reflection is suppressed by titania’s bandgap absorbance. This amplification effect has been recently identified for co-assembled TiO_2_ PCs opals on SA degradation under UV-Vis light [33], in perfect agreement with the present results for the unmodified photonic films in Figure 5d. A further shift of the PC’s stopband to the visible range by the increase of macropore size such as PC330, leads to the drop of photocatalytic activity due to the absence of any electronic absorption as well as the decrease of the films’ surface area, which in the case of self-assembled TiO_2_ inverse opals is largely determined by the mesopores in the nanocrystalline skeleton walls that increase for the smaller macropore diameters [59]. On the other hand, the bare mesoporous P25 films exceeded significantly the PCs’ performance, mainly due to the presence of rutile nanocrystals, whose narrower electronic bandgap of about 3.0 eV and the interfacial charge transfer from rutile to the anatase nanoparticles underlie their VLA photocatalytic activity [71].

FeO_x_ surface modification resulted invariably in marked improvements of *r*_Vis_ for all CC-modified TiO_2_ films (Figure 5b), especially after the 1st CC cycle, with the inverse opals outperforming the benchmark mesoporous FeO_x_−P25 films (by about 45% after the 1st CC cycle) in spite of their higher catalyst mass. Moreover, the obtained *r*_Vis_ rates depended distinctively on the macropore diameter of the inverse opals (Figure 5b), indicating significant differences in the light-harvesting ability of the PC films. Specifically, FeO_x_–PC260-1st exhibited the highest *r*_Vis_, followed closely by FeO_x_-–C220-1st, whereas the larger diameter FeO_x_–PC330-1st presented the lowest activity (by about 75%). This size dependence can be closely associated with the PC’s stopband variation and the concomitant slow photon amplification effects, most prominent for PC260, whose stopband is expected at ~393 nm in water (Table 1). Assuming that the PBG’s spectral width is about the FWHM ≈ 30 nm of the Bragg R% peak (Figure 1c) [62], the PC260′s stopband in water is expected at 393 ± 15 nm and the corresponding red-edge slow photons, which span a narrower spectral range of about 20 nm [18], will occur roughly within 410–430 nm, matching closely the electronic absorption of FeO_x_ nanoclusters (Figure S5). This behavior was directly identified by R% measurements for the FeO_x_–PC260-1st films in water (Figure 4e), where the red-shift of the specular R% peak at λ_exp_ (15°) by about 60 cm^−1^ was directly confirmed complying with the predictions of modified Bragg’s law (Table 1). A larger red-shift of the broad DR% peak in water from about 350 (air) to 420 cm^−1^ (water) was also observed (Figure 4f) reflecting closely the spectral shift of the red-slow photon region that matches the FeO_x_ electronic absorption range. The slow photon region is expected to blue-shift by about 20 nm for PC220 (Table 1), i.e., in the range of 390–410 nm, leading to a smaller spectral overlap with FeO_x_ absorbance at λ > 400 nm, applied for the visible light photocatalytic experiments. On the other hand, no appreciable slow-photon effects are expected for the larger diameter FeO_x_−PC330-1st, whose stop band in water at about 517 nm (Table 1) is well above FeO_x_ absorbance after the 1st CC cycle for appreciable blue-edge slow photon effects.

To investigate the oxidation power of photogenerated holes in the visible light degradation mechanism of SA, comparative photocatalytic tests were performed for the best performing FeO_x_−PC260-1st in the presence of IPA and formic acid as hydroxyl radical and hole scavengers, respectively (Figure 5c). In the former case, a relatively small decrease of *r*_Vis_ by 16% was observed, while a marked drop by 61% was detected in the case of formic acid, indicating that holes generated in the FeO_x_–TiO_2_ films under visible light are the main reactive oxygen species for SA decomposition. However, an increase of FeO_x_ loading for both PC260 and P25 films after consecutive 2nd and 3rd cycles resulted in the systematic drop of *r*_Vis_ (Figure 5b), despite the increase of visible light absorbance detected in the corresponding DR% spectra (Figure 5b,d). This behavior may be partly associated with the formation of FeO_x_ aggregates on the nanocrystalline anatase walls (Figure 2h) that will impair the FeO_x_–TiO_2_ interfacial charge transfer as well as the suppression of photonic enhancement effects by the increased FeO_x_ absorption that impedes light propagation in the PC structure [72]. This detrimental effect was significantly aggravated in the case of UV-Vis illumination (Figure 5d), where FeO_x_ surface modification resulted in the decrease of r_UV-Vis_ from the 1st cycle for the PCs, followed by a continuous drop for subsequent CC cycles. A small increase of r_UV-Vis_ was detected for the FeO_x_–P25 films (by about 10%) after the 1st CC cycle, followed by an appreciable decrease after the 2nd and 3rd CC cycles. This behavior indicates that besides absorption losses for higher FeO_x_ loading that inhibit photonic amplification effects and deteriorate the VLA photocatalytic performance of PC films, additional reasons underlie the decline of photocatalytic performance in the FeO_x_–TiO_2_ system. In fact, similar behavior was earlier reported for Fe_x_O_y_–P25 nanocomposites [53], where, the presence of low amounts of isolated Fe(III)-oxo clusters on the surface of TiO_2_ nanoparticles resulted in significant photocatalytic activity and hydroxyl radical formation under visible light. However, the UV photocatalytic rates were comparatively lower than those of the pristine P25 nanoparticles, indicating that Fe(III) oxo-centers on TiO_2_ may act as trapping sites that moderate the composite’s oxidation power.

### 3.3. Charge Separation

**Figure 6:**
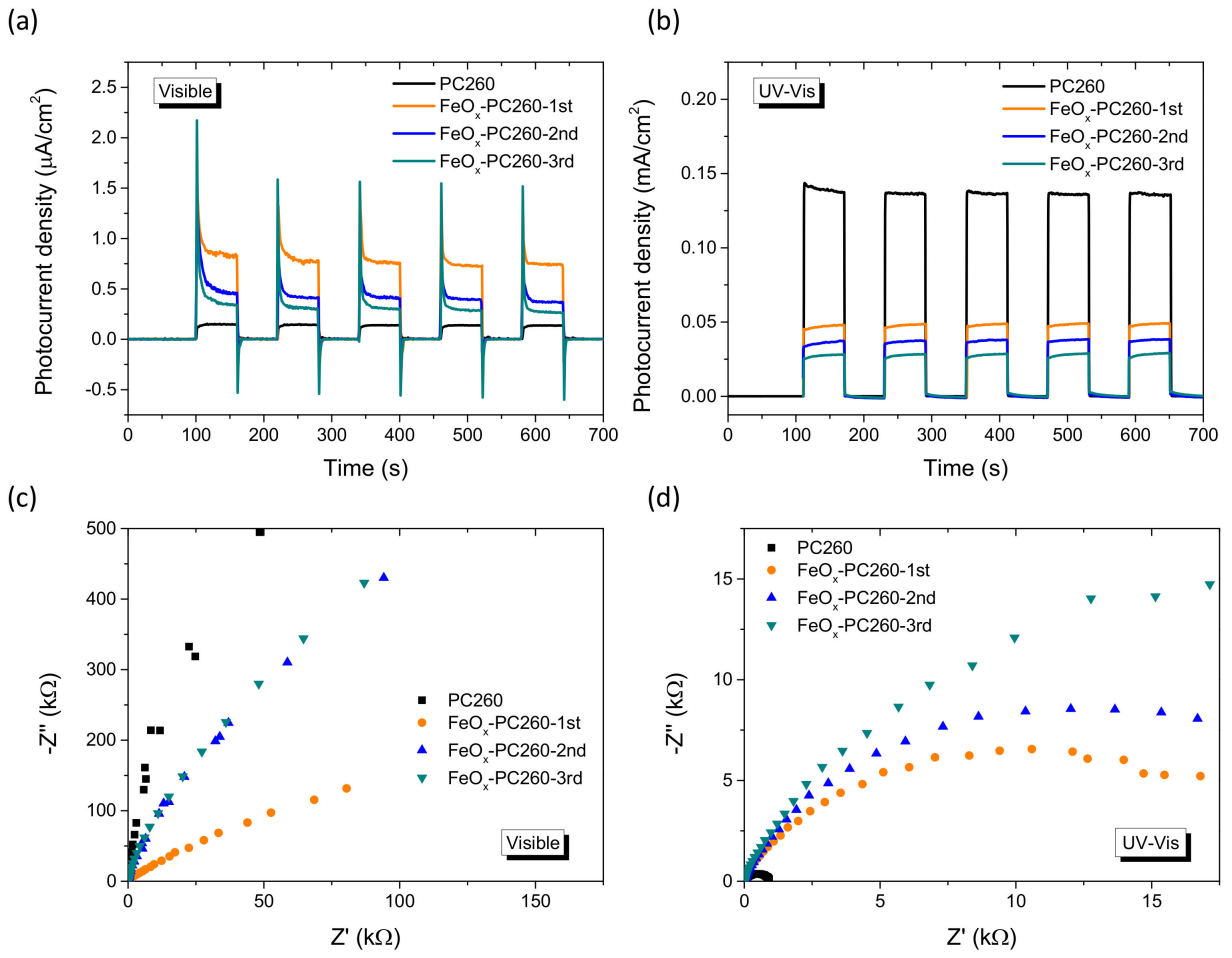
Transient photocurrents under chopped light and EIS Nyquist plots for (**a**,**c**) visible and (**b**,**d**) UV-Vis illumination for the FeO_x_–PC260 films.

To investigate charge separation in the FeO_x_−PC films, photoelectrochemical measurements were performed under visible and UV-Vis irradiation. Photocurrent transients for the CCC-modified PC260 films under chopped visible light illumination at 0.2 V vs. Ag/AgCl (Figure 6a) showed an appreciable increase for both the instantaneous (hole current) and steady-state photocurrent for FeO_x_–PC260-1st compared to the pristine PC260, reflecting the enhanced visible light-harvesting of the modified PC films for low FeO_x_ contents. However, the photocurrent densities decreased progressively for FeO_x_–PC260-2nd and FeO_x_–PC260-3rd despite their higher visible light absorbance caused by the increase of FeO_x_ loading. This behavior, which mirrors closely the corresponding variation of the *r*_Vis_ rates on SA photodegradation, indicates that surface recombination contributes largely to the films’ performance decrease, especially after the 2nd and 3rd CC cycles [73]. 

On the other hand, the photocurrents decreased continuously with FeO_x_ loading under UV-Vis irradiation (Figure 6b), pointing to reduced UV oxidation power of FeO_x_−PC films, alike SA photodegradation experiments. This effect was supported by EIS measurements on the FeO_x_–PC260 films under both visible and UV–Vis irradiation. Comparative Nyquist plots (Figure 6c) showed that the radius of the capacitive arc at higher frequencies in the EIS plane decreased for FeO_x_–PC260-1st compared to the pristine PC260 and increased after the 2nd and 3rd CC cycles under visible light, indicative of reduced charge transfer resistance for the 1st CC cycle. On the other hand, the opposite behavior was observed under UV-Vis light, where impedance and the corresponding arc radius increased consecutively for the FeO_x_–PC260 films, indicating that FeO_x_ nanoclusters on the nanocrystalline TiO_2_ inverse opal walls may create surface traps that compromise the materials’ oxidation power under UV-Vis light. 

**Figure 7:**
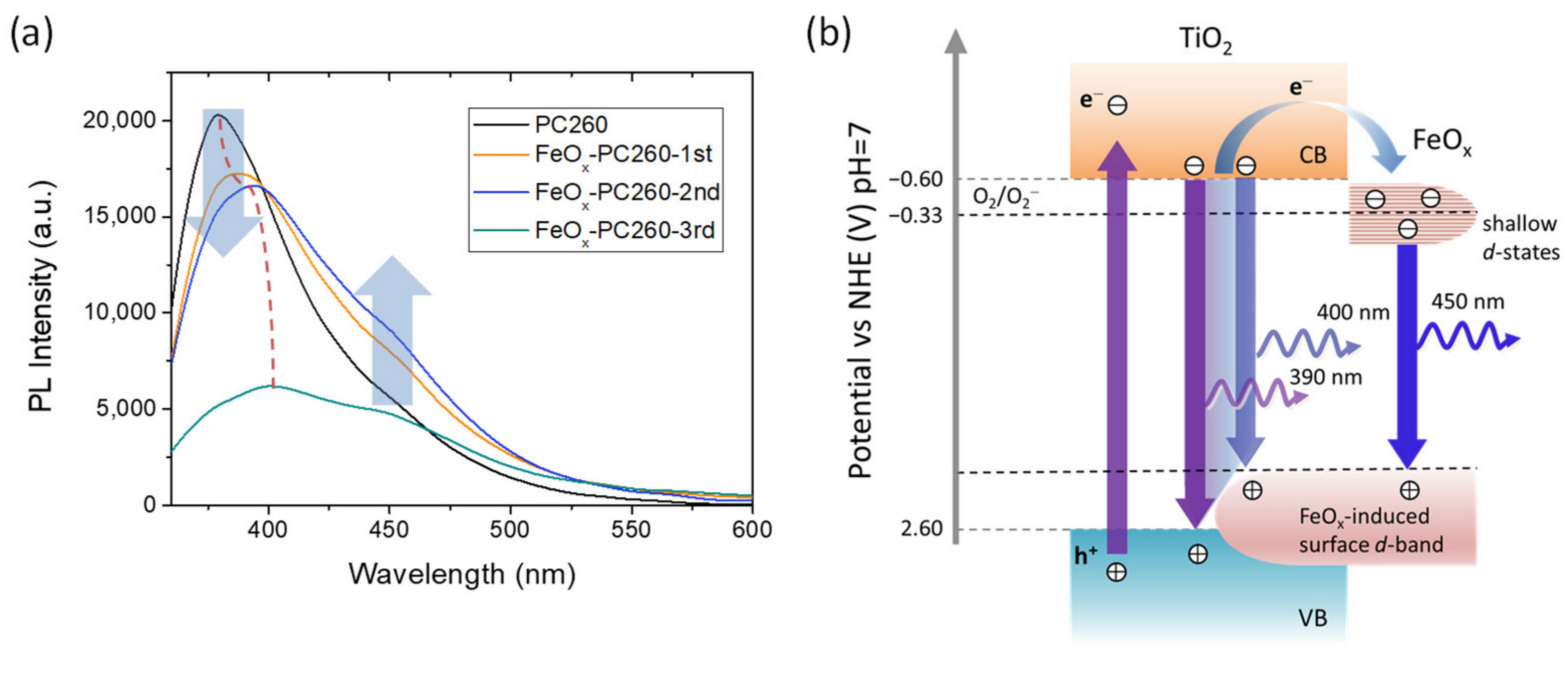
(**a**) PL spectra of the CCC-modified PC260 films and (**b**) schematic depicting the emission processes in FeO_x_–PC under UV excitation. The anatase band edge positions were taken from [76].

Charge recombination and the presence of defect states were explored by PL spectroscopy for the FeO_x_–PC260 films under 275 nm excitation, as shown in Figure 7a. The PL spectrum of pristine PC260 showed a broadband at 380 nm arising from the near-band gap emission via indirect band-to-band transitions of anatase nanoparticles, accompanied by a much weaker shoulder at about 450 nm originating from shallow defect states due to oxygen vacancies [5,74,75]. Surface modification by FeO_x_ nanoclusters resulted in the progressive decrease of near-band gap emission along with a red shift of its spectral position reaching 400 nm for FeO_x_–PC260-3rd. These features comply favorably with the interfacial electron transfer and the rise of the composite’s valence band (VB) maximum by means of a FeO_x_−induced surface d-band above the anatase valence band edge, respectively (Figure 7b). In addition, the broad defect-induced PL emission at 450 nm was gradually enhanced for the FeO_x_−PC260 films after the 1st and 2nd CC cycles, indicating the increase of radiative recombination via shallow FeO_x_−induced states below the anatase conduction band (CB) edge [56,57]. Such vacant surface d-states have been shown to lie close to those of pristine anatase and the reduction potential of O_2_/O_2_^−^ (E^0^ = −0.33 V vs. NHE) allowing superoxide radical formation by trapped photogenerated electrons without impeding the photocatalytic activity of CCC-modified TiO_2_ under optimal loading of FeO_x_ nanoclusters [37]. However, a broad distribution of these defect states can be assumed in the present case because of the mixed-phase composition of FeO_x_ nanoclusters (Figure 7b), which will be further expanded with the increase of FeO_x_ loading and aggregation. Defect states at variable energies straddling the O_2_/O_2_^−^ reduction potential may then increase surface recombination under UV-Vis excitation, since trapped electrons at states more negative than E^0^(O_2_/O_2_^−^) will recombine resulting in the observed increase of defect-induced PL emission (Figure 7b) and the higher recombination kinetics probed by EIS under UV-Vis light. On the other hand, the persistent recombination via these defect states under visible light, cannot completely suppress charge separation in the FeO_x_–TiO_2_ system that sustains appreciable VLA photocatalytic performance at low FeO_x_ loading amounts, which is further assisted by visible light trapping in the PBG engineered inverse opals.

## 4. Conclusions

Surface modification of co-assembled TiO_2_ inverse opals by variable amounts of FeO_x_ nanoclusters was implemented by successive chemisorption-calcination cycles of Fe(III) acetylacetonates on the nanocrystalline anatase PC walls, leading to composite FeO_x_–TiO_2_ photonic films with extended visible light absorption. PBG engineering of the co-assembled inverse opals across the composite FeO_x_–TiO_2_ absorption edge was performed by tuning the templating colloidal spheres size. Distinctive diameter-dependent improvements of the SA photocatalytic degradation rates and photocurrent density were thus obtained under visible light for low loading amounts of FeO_x_ nanoclusters, which outperformed benchmark P25 films subjected to the same treatment. Scavenger experiments indicated that holes generated in the FeO_x_–TiO_2_ films under visible light are the major reactive oxygen species for SA decomposition, evading the weak oxidizing power of photogenerated holes frequently met in VLA TiO_2_ photocatalysts. The observed performance amplification was related to the improved charge separation induced by the interfacial coupling between TiO_2_ and FeO_x_ in combination with optimal light trapping by red-edge slow photons resonating with the FeO_x_–TiO_2_ absorption edge. However, an increase in FeO_x_ loading resulted in the drastic drop of photocatalytic performance mainly because of a broad distribution of defect d-states straddling the O_2_/O_2_^−^ reduction potential that increase surface recombination according to photoelectrochemical and PL experiments. Fine-tuning of heterostructured metal oxide PC photocatalysts’ composition is concluded to be a key factor for the fabrication of efficient VLA photocatalytic films with enhanced visible light trapping and charge separation.

## Figures and Tables

**Table 1:** Optical parameters for the TiO_2_ inverse opal films.

Sample	*D*^a^ (nm)	*λ*_exp_ (15°) ^b^ (Air)	*n*_eff_ (Air)	1-*f*	*n*_eff_ (H_2_O)	*λ*(0°) ^c^ (Air)	*λ*(0°) ^c^ (H_2_O)
PC220	135	325	1.50	0.22	1.68	330	371
PC260	150	335	1.39	0.17	1.60	341	393
PC330	200	437	1.36	0.16	1.58	445	517

^a^*D* = inverse opal macropore diameter determined from SEM. ^b^ *λ*_exp_(15°) = stop band wavelength determined from R% spectra at 15° incidence angle. ^c^ *λ*(0°) = stop band wavelength according to the modified Bragg law at 0° incidence angle.

## Data Availability

Not applicable.

## Supplementary Materials

The following are available online at https://www.mdpi.com/article/10.3390/ma14237117/s1, Figure S1: Top view SEM images of pristine and FeO_x_ modified PC260 and P25 films by two consecutive CC cycles of Fe(acac)_3_, Table S1: Elemental EDX analysis of the FeO_x_—modified PC and P25 films, Figure S2: Raman spectra for the pristine and CCC modified PC and P25 films at 514 nm, Figure S3: Raman spectra for the FeO_x_–P25 films (thick lines) at 514 nm before and after local annealing experiments, Figure S4: Reference Raman spectra of representative iron oxides at 514 nm and Raman spectra acquired on maghemite (γ-Fe_2_O_3_) nanoparticles after local laser annealing experiments at variable laser power densities, Figure S5: Kubelka-Munk transform function F(R) obtained from the DR (%) of the FeO_x_–P25 films, Figure S6: SA absorbance spectra in the presence of FeO_x_–PC260-1st and SA photodegradation kinetics under visible and UV-Vis light illumination for the pristine and FeO_x_—modified PC and P25 films.
