# Supplementary material for: Visible Light Trapping against Charge Recombination in FeOx–TiO2 Photonic Crystal Photocatalysts
Source: Materials (Basel). 2021 Nov 23;14(23):7117. doi: 10.3390/ma14237117 (PMC8658129; doi:10.3390/ma14237117)
Supplement: Supplementary file 1 [file materials-14-07117-s001.zip › materials-1452691-Supplementary materials rev (1).pdf]

materials

Supplementary Materials

Visible Light Trapping against Charge Recombination in FeO_x–TiO_2 Photonic Crystal Photocatalysts

Martha Pylarinou [1], Alexia Toumazatou [1], Elias Sakellis [2], Evangelia Xenogiannopoulou [2], Spiros Gardelis [1], Nikos Boukos [2], Athanasios Dimoulas [2] and Vlassis Likodimos [1,*]

1 Section of Condensed Matter Physics, Department of Physics, National and Kapodistrian University of Athens, University Campus, 15 784, Greece
2 Institute of Nanoscience and Nanotechnology, National Center for Scientific Research "Demokritos", 15341 Agia Paraskevi, Athens, Greece
* Correspondence: vlikodimos@phys.uoa.gr; Tel.: +30 2107276824

S1. PBG determination from specular reflectance

The stop band positions were estimated by modified Bragg's law for first order diffraction from the (111) fcc planes [1]:

$$\lambda = 2d_{111}\sqrt{n_{eff}^2 - \sin^2\theta},$$

where λ is the stop band wavelength, $d_{111} = \sqrt{\frac{2}{3}}\ D$ is the spacing between (111) planes, D is the macropore diameter and $n_{eff}^2 = n_{\mathrm{void}}^2 f + n_{\mathrm{TiO_2}}^2(1-f)$ is the volume-weighted average of the void spheres' refractive index, n_{void}, and titania $n_{\mathrm{TiO_2}}$, occupying the inverse opal frame, with f being the filling fraction ($f = 0.74$ for the ideal fcc lattice) and θ the angle between the incident beam and the plane normal. Using the experimental stop band wavelengths at $\theta = 15^{\mathrm{o}}$ along with the macropore diameters determined by SEM (Table 1) for $n_{\mathrm{TiO_2}} = 2.55$ and $n_{\mathrm{air}} = 1.0$, the n_{eff} values and filling fractions $(1-f)$ were determined in air, as summarized in Table 1. In addition, using the obtained filling fractions and $n_{\mathrm{H_2O}} = 1.33$, the stop band positions were estimated for the PC films in water, where the photocatalytic reaction takes place (Table 1).

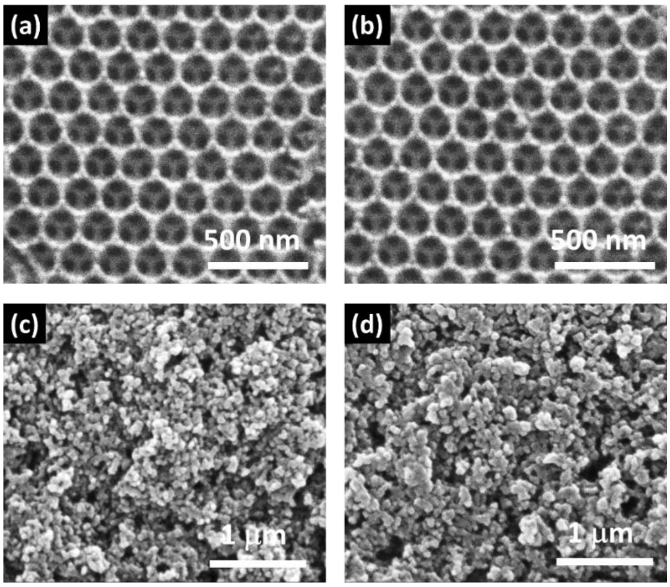

Figure S1. Top view SEM images of PC260 and P25 films (a), (c) before and (b), (d) after surface modification by two consecutive CC cycles of $Fe(acac)_3$.

Table S1. Elemental EDX analysis of the FeO_x-modified PC and P25 films.

Films	Ti (at %)	Fe (at %)	Fe /Ti
FeO_x-PC220-1st	96,19	3,82	0,040
FeO_x-PC330-1st	96,49	3,51	0,036
FeO_x-PC260-1st	96,44	3,56	0,037
FeO_x-PC260-2nd	93,80	6,20	0,066
FeO_x-PC260-3rd	90,69	9,31	0,103
FeO_x-P25-1st	97,26	2,74	0,028
FeO_x-P25-2nd	94,15	5,85	0,062
FeO_x-P25-3rd	90,26	9,74	0,108

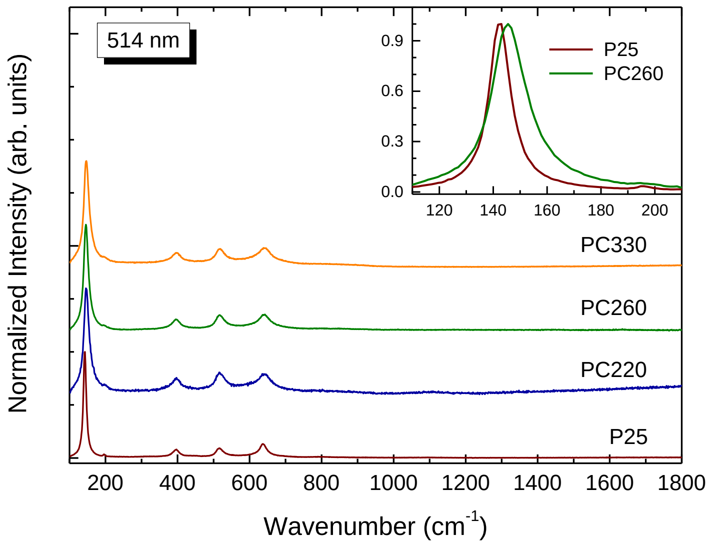

Figure S2. Raman spectra for the pristine and CCC modified PC and P25 films at 514 nm. The inset compares the lowest frequency anatase E_g mode for the PC260 and P25 films.

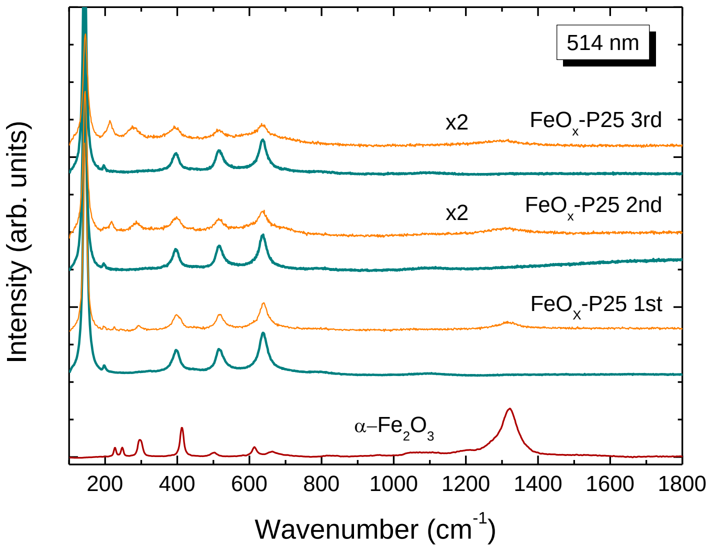

Figure S3. Raman spectra for the FeO_x-P25 films (thick lines) at 514 nm. Thin lines display the corresponding Raman spectra after local annealing experiments performed by focusing the 514 nm laser beam by the 100× objective at full laser power (2.5 mW) for 60 s on the films followed by normal acquisition on the same spot at low laser power (0.05 mW/μm^2). The reference spectrum of nanocrystalline hematite *a*-Fe_2O_3 powder is shown for comparison at 514 nm.

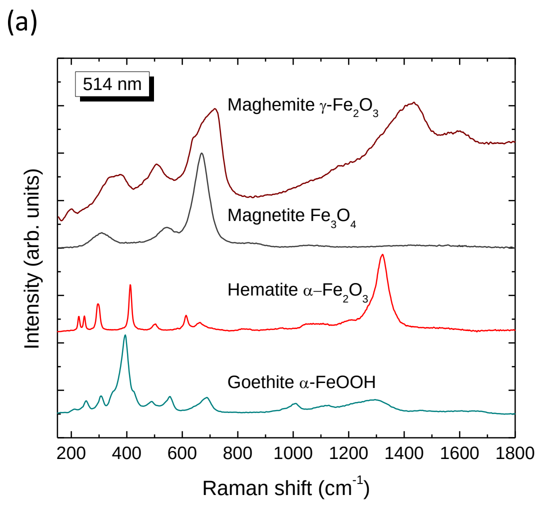

Figure S4. (a) Reference Raman spectra of representative iron oxides at 514 nm. (b) Raman spectra acquired on maghemite (γ-Fe_2O_3) nanoparticles after local laser annealing experiments at variable densities of the full laser power (2.5 mW) for 60 s.

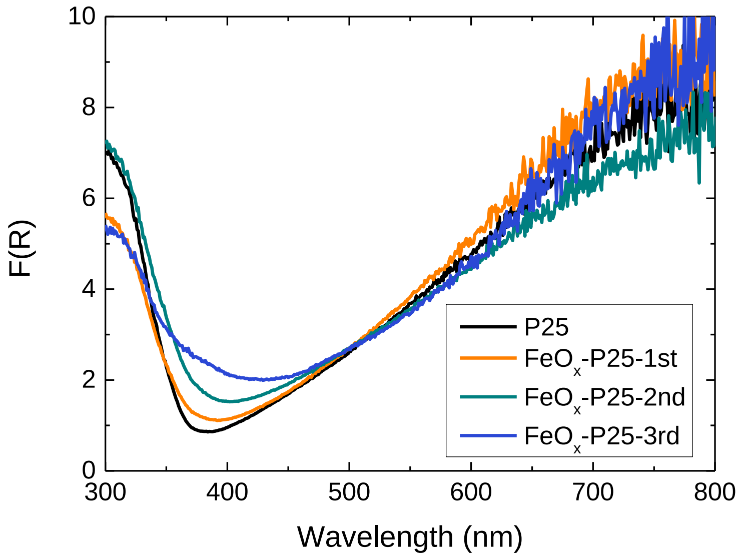

Figure S5. Kubelka-Munk transform function F(R) obtained from the DR (%) of the FeO_x-P25 films.

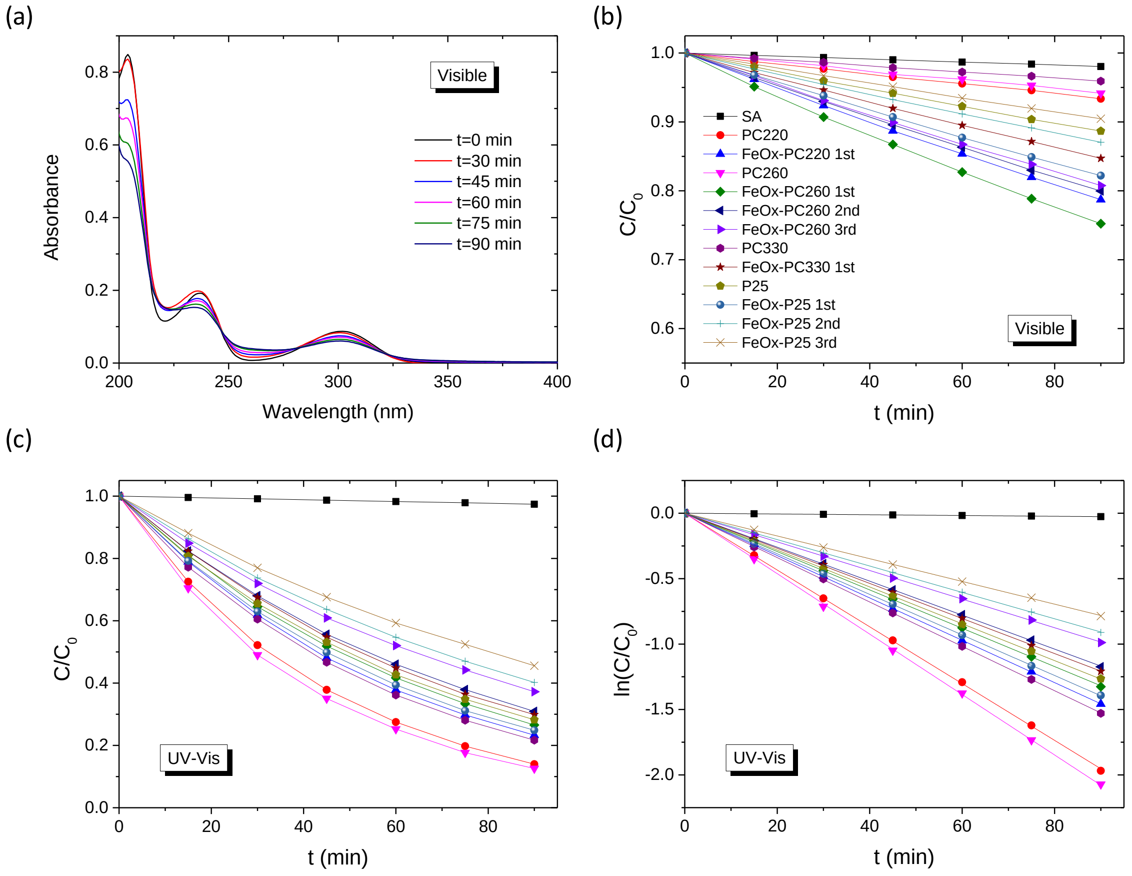

Figure S6. (a) SA absorbance spectra in the presence of FeO_x-PC260-1st and SA photodegradation kinetics under (b) visible and (c), (d) UV-Vis light illumination for the pristine and FeO_x-modified PC and P25 films.

References

1. Stein, A., Wilson B.E., Rudisill S.G. Design and functionality of colloidal-crystal-templated materials-chemical applications of inverse opals. *Chem. Soc. Rev.* **2013**, *42*, 2763–2803. https://doi.org/10.1039/c2cs35317b.
